# Slow cortical potential and theta/beta neurofeedback training in adults: effects on attentional processes and motor system excitability

**DOI:** 10.3389/fnhum.2014.00555

**Published:** 2014-07-24

**Authors:** Petra Studer, Oliver Kratz, Holger Gevensleben, Aribert Rothenberger, Gunther H. Moll, Martin Hautzinger, Hartmut Heinrich

**Affiliations:** ^1^Department of Child and Adolescent Mental Health, University Hospital of ErlangenErlangen, Germany; ^2^Child and Adolescent Psychiatry, University Medical Center GöttingenGöttingen, Germany; ^3^Department of Clinical Psychology, Institute of Psychology, Eberhard Karls University TübingenTübingen, Germany; ^4^Heckscher-Klinikum, MünchenGermany

**Keywords:** neurofeedback, slow cortical potential (SCP) training, theta/beta training, event-related potentials (ERPs), transcranial magnetic stimulation (TMS), contingent negative variation (CNV)

## Abstract

Neurofeedback (NF) is being successfully applied, among others, in children with attention deficit/hyperactivity disorder (ADHD) and as a peak performance training in healthy subjects. However, the neuronal mechanisms mediating a successful NF training have not yet been sufficiently uncovered for both theta/beta (T/B), and slow cortical potential (SCP) training, two protocols established in NF in ADHD. In the present, randomized, controlled investigation in adults without a clinical diagnosis (*n* = 59), the specificity of the effects of these two NF protocols on attentional processes and motor system excitability were to be examined, focusing on the underlying neuronal mechanisms. Neurofeedback training consisted of 10 double sessions, and self-regulation skills were analyzed. Pre- and post-training assessments encompassed performance and event-related potential measures during an attention task, and motor system excitability assessed by transcranial magnetic stimulation. Some NF protocol-specific effects have been obtained. However, due to the limited sample size medium effects did not reach the level of significance. Self-regulation abilities during negativity trials of the SCP training were associated with increased contingent negative variation amplitudes, indicating improved resource allocation during cognitive preparation. Theta/beta training was associated with increased response speed and decreased target-P3 amplitudes after successful theta/beta regulation suggested reduced attentional resources necessary for stimulus evaluation. Motor system excitability effects after theta/beta training paralleled the effects of methylphenidate. Overall, our results are limited by the non-sufficiently acquired self-regulation skills, but some specific effects between good and poor learners could be described. Future studies with larger sample sizes and sufficient acquisition of self-regulation skills are needed to further evaluate the protocol-specific effects on attention and motor system excitability reported.

## INTRODUCTION

During neurofeedback (NF) training individuals learn to acquire self-regulation skills of particular brain activity patterns by receiving positive feedback on brain activity changes in the desired direction. The rationale of NF is derived from observations that a specific mental state (e.g., attention) is associated with a certain brain state (e.g., more pronounced beta activity). Thus, by training to acquire a specific brain state, NF aims at enhancing the mental state associated with this brain state, and thereby improving behavioral self-regulation in daily life situations (Gevensleben et al., 2012; Moriyama et al., 2012).

A whole variety of NF protocols has been developed in order to target different mental states and associated behavior. Two basic types of NF protocols can be distinguished: frequency band training and training of slow cortical potentials (SCPs).

In a frequency band training, a decrease and/or increase of the amplitudes of specific encephalogram (EEG) frequency bands are rewarded. One established frequency band training is the theta/beta training which aims at enhancing a state of sustained attention by reinforcing reductions in theta (4–8 Hz) and increases in beta (13–20 Hz) amplitudes^1^ recorded at the vertex (Cz).

A training of SCPs (SCP training) is based on recordings of SCPs at the vertex, which last from several hundred milliseconds to several seconds and which are related to the level of excitability of the underlying cortical areas (Birbaumer et al., 1990; Heinrich et al., 2007). Surface-negative SCP shifts reflect increased excitation of the underlying cortical areas and typically occur during behavioral and cognitive preparation. Surface-positive SCP shifts are related to decreased excitation and are observed among others during behavioral inhibition. During SCP training, participants learn to change between an activated/attentive state and a deactivated/relaxed state by modulating their SCPs toward more negative and positive amplitudes, respectively.

The NF protocols described above have been applied both in clinical and peak performance domains. Neurofeedback in clinical domains targets reducing clinical symptomatology in patients, with one main application in children with attention deficit/hyperactivity disorder (ADHD). Neurofeedback as a peak performance training is applied in healthy persons with the aim of further enhancing already good performance.

In children with ADHD, for both theta/beta and SCP training positive effects on reducing clinical symptomatology (inattention, hyperactivity/impulsivity) and improving cognitive performance have been reported (for review, see, e.g., Mayer et al., 2012b; Moriyama et al., 2012; Arns et al., 2014), and with especially more recent studies being based on randomized-controlled designs (e.g., Drechsler et al., 2007; Gevensleben et al., 2009; Duric et al., 2012; Meisel et al., 2013; Steiner et al., 2014). In the so far largest NF study in ADHD which included both theta/beta and SCP NF training, the effectiveness of these NF protocols in ADHD has been shown (Gevensleben et al., 2009). A recent meta-analysis indicated the effectiveness of both theta/beta and SCP training protocols in children with ADHD (Arns and Strehl, 2013), even though currently there is a controversial discussion on the effectiveness of NF in ADHD (Lofthouse et al., 2012; Sonuga-Barke et al., 2013; Arns et al., 2014). In recent review articles NF, especially theta/beta and SCP NF, was concluded to be a clinically effective treatment in ADHD (Arns et al., 2014) and the importance of gaining further insights on the underlying mechanisms of action as well as on disentangling specific from non-specific effects was stressed (Gevensleben et al., 2012; Moriyama et al., 2012; Arns et al., 2014).

In the peak performance domain, so far NF studies were mainly conducted in adult participants (for a comprehensive review, see Gruzelier, 2013). Overall, theta/beta and SCP protocols are less well established in the peak performance domain compared to the field of ADHD, but some results have been published. Theta/beta training protocols were observed to enhance arousal (Egner and Gruzelier, 2004), but not musical performance (Egner and Gruzelier, 2003). SCP training was reported to exert positive effects on response speed during “negativity” trials (Birbaumer et al., 1990; Birbaumer, 1999).

So far, more commonly applied protocols in the peak performance domain comprise, among others sensorimotor rhythm (SMR) training as well as alpha/theta training. Sensorimotor rhythm training was reported to enhance semantic working memory (Vernon et al., 2003), sustained attention (Egner and Gruzelier, 2004), microsurgical skills (Ros et al., 2009), reaction times (RTs), and spatial rotation abilities (Doppelmayr and Weber, 2011). However, no positive effects of SMR training were observed for the D2 attention test (Doppelmayr and Weber, 2011), for creativity (Doppelmayr and Weber, 2011), and for musical performance (Egner and Gruzelier, 2003). Alpha/theta training has been observed to enhance, e.g., musical performance (Egner and Gruzelier, 2003; Gruzelier, 2009, Gruzelier et al., 2013a), and cognitive creativity (Gruzelier et al., 2013b), as well as to enhance dance performance in one study (Raymond et al., 2005a) but not in another (Gruzelier et al., 2013b).

Overall, positive effects of different NF protocols have been reported both for their clinical application, e.g., in children with ADHD, as well as for different applications (e.g., attention, performing arts) in the peak performance domain. But despite the evergrowing diversity of NF protocols and their applications, mechanisms mediating a successful NF training are still not completely understood.

In order to study the mechanisms underlying the treatment effects of different NF protocols, especially more recent NF studies have employed neurophysiological measures like event-related potentials (ERPs), and one study has applied transcranial magnetic stimulation (TMS; Ros et al., 2010). The rationale for applying these methods in NF studies is derived from the association of specific brain electrical activity to distinct mental states and behavior (Moriyama et al., 2012).

ERP components, such as the P3 and the contingent negative variation (CNV) are related to cognitive stimulus processing stages (Banaschewski and Brandeis, 2007) and have been used to study covert attention, e.g., in ADHD and NF research. The P3 is thought to reflect attentional resource allocation, stimulus evaluation as well as context updating processes (Banaschewski and Brandeis, 2007; Polich, 2007). In adults, an increase in P3 amplitude has been revealed after a combined beta1 (15–18 Hz) and SMR (12–15 Hz) training (Egner and Gruzelier, 2001), and in a later study after a beta1 but not after an SMR training reflecting increased activation in an attentional alertness network (Egner and Gruzelier, 2004). In children with ADHD, no increase in P3 amplitude was revealed after a combined SCP and theta/beta training (Wangler et al., 2011). The CNV, a negative polarization of an SCP occurring between a warning and a target stimulus, reflects attentional processes related to anticipation and preparation (Birbaumer et al., 1990). Increased CNV amplitudes, have been observed after SCP training in children with ADHD (Heinrich et al., 2004; Wangler et al., 2011) and according to preliminary results also in adults with ADHD (Mayer et al., 2012a,b) indicating improved resource allocation.

Transcranial magnetic stimulation allows investigating excitatory mechanisms of the motor system (Reis et al., 2008), and to distinguish processes of short-interval intracortical inhibition (SICI) and intracortical facilitation (ICF; Kujirai et al., 1993). One study has examined the effects of a single session of NF (alpha suppression or low beta enhancement) in healthy adults on corticomotor excitability by means of TMS (Ros et al., 2010). Based on a non-conservative statistical analysis, this study provided hints for decreased SICI after an alpha, but not after a low beta training.

Self-regulation ability is a measure assessing changes in the trained EEG parameters in the course of NF training and is considered to mediate effects of NF on behavior. In addition, associations of learned self-regulation of a distinct EEG parameter with improvements in outcome measures can provide evidence for specific effects of different NF protocols (Gruzelier, 2013). So far, self-regulation has more consistently been examined in peak performance (for a review, see Gruzelier, 2014) than in clinical studies.

The aim of the present randomized controlled investigation in “healthy” adult participants was to examine the specificity of the effects of a theta/beta, and an SCP training on attention as well as on motor system excitability. The focus of the study was to gain further insights into the neurophysiological mechanisms underlying these two NF training protocols by also assessing ERP (P3 and CNV) and TMS (SICI and ICF) measures.

Regarding attention, on the performance level, larger training-related increases in attention were expected for the two NF groups compared to the control group, while no differential effects were expected between theta/beta and SCP protocols. At the level of ERP measures, pre–post increases were expected to be larger in the two NF groups compared to the control group. The largest pre–post increase in P3 amplitude was expected after theta/beta training, and the largest increase in CNV amplitude after SCP training. Good self-regulation skills during theta/beta and SCP training were expected to be associated with pre–post P3 amplitude changes and with a larger pre–post increased in CNV amplitude, respectively.

In comparison to the control group, theta/beta and SCP training were expected to have effects on motor system excitability. As the present study was the first examination of motor system excitability by means of TMS after a complete NF training, we had no directed hypotheses regarding differential effects of the two NF training protocols on SICI and ICF.

## MATERIALS AND METHODS

### PARTICIPANTS

Fifty-nine subjects (aged 19–31 years) participated in this randomized, controlled study. Exclusion criteria were: a psychiatric or neurologic diagnosis, a cardiovascular disease, a pathological EEG or ECG, pregnancy, estimated IQ below 80 (based on the Verbal Comprehension Index and the Perceptual Reasoning Index of the German version of the Wechsler Adult Intelligence Scale), values above norm values of the Symptom Checklist-90-R: SCL-90-R (Derogatis and Savitz, 2000). Two subjects dropped out of the study due to schedule problems directly after the pre-assessments, one subject had to be excluded due to German-language difficulties and one subject due to a personal crisis which occurred in the course of training. Thus, the final sample comprised 55 adults who have completed the study.

These participants were randomly assigned (randomized list without any stratification) to one of three groups: theta/beta frequency band training (T/B: *n* = 19), training of SCPs (SCP: *n* = 19), or control training (CON: *n* = 17). **Table 1** provides an overview over the demographic and psychological characteristics of the final sample.

**Table 1:** Demographic and psychological characteristics of the sample.

	Age (years)	Sex m/f	Estimated IQ	SCL-90: GSI
T/B (*n* = 19)	24.62 ± 2.56	7/12	105.95 ± 6.19	0.23 ± 0.18
SCP (*n* = 19)	25.08 ± 2.47	10/9	105.24 ± 7.67	0.14 ± 0.10
CON (*n* = 17)	23.59 ± 3.06	7/10	103.65 ± 9.31	0.33 ± 0.20
ANOVA	*F*(2,52) = 1.33, n.s.	*F*(2,52) = 0.49, n.s.	*F*(2,52) = 0.41, n.s.	*F*(2,51) = 3.62, *p* < 0.05

*For each group demographic and psychological characteristics are depicted (mean value and standard deviation). T/B, theta/beta frequency band training group; SCP, SCP training group; CON, control training group; m, male; f, female. Estimated IQ: based on the Verbal Comprehension Index and the Perceptual Reasoning Index of the German version of the Wechsler Adult Intelligence Scale. GSI, Global Severity Index of the Symptom-Checklist-90-R (SCL-90) self-report measure. ANOVA, analysis of variance with the between-subject factor GROUP. Significant effects for the GSI were related to higher scores in the CON group.*

Written informed consent was obtained from all participants. The experiment was conducted in accordance with the Declaration of Helsinki and was approved by the Ethics Committee of the Medical Faculty of the University Hospital of Erlangen.

### DESIGN

All trainings including pre- and post-assessments were conducted in the Department of Child and Adolescent Mental Health at the University Hospital of Erlangen. The participation in the study extended for about 2 months per person and participants received an expense allowance. All three training programs were administered by the same trainers.

#### Neurofeedback

The two NF trainings (T/B and SCP) consisted of 20 sessions à 50 min each which were conducted as 10 double sessions mostly taking place twice per week. Visual feedback information was provided. Both theta/beta and SCP training included about 40% transfer trials during which participants received no feedback about their current brain state. Subjects in the T/B and SCP groups were instructed to develop individual (intuitive or cognitive) strategies in order to achieve the desired brain state. Starting with the fifth double session, subjects of both NF groups applied their strategies to attention-demanding tasks (in turn a game of darts or a continuous performance test) in the last 10 min of a double session – as a first step toward a transfer to other relevant situations. Moreover, participants were instructed to practice the transfer of their strategies at least once each day in daily life situations in which they wanted to improve their attention or well-being.

During theta/beta self-regulation blocks, subjects were asked to reduce their theta activity (4–8 Hz) and simultaneously increase their beta activity (13–20 Hz) relative to a baseline assessed at the beginning of a training session and received feedback by means of changing bars which had to be reduced and increased, respectively. The aim was to achieve an attentive but relaxed state. To calculate theta and beta activity, Buttworth filters (48 dB/octave) were applied and feedback information was determined 10 times per second by means of a moving time window of 2 s length. In the first few training sessions, most self-regulation blocks lasted for 5 min, while in the course of training, self-regulation blocks were extended to 10 min in order to train staying focused for a longer time period.

During SCP training, feedback was provided in the form of a ball that subjects were to direct upwards in negativity trials and downwards in positivity trials (equal number of positivity and negativity trials, randomized order). A trial lasted for 8 s and consisted of a 2 s baseline period and a 6 s feedback period (intertrial interval: 5 ± 1 s). Training was performed in blocks of 40–60 trials. The training aimed at enhancing an activated / attentive state during negativity trials as well as a deactivated/relaxed state during positivity trials. Feedback was provided based on the mean SCP amplitude based on a moving time window of 1 s length which was calculated 10 times per second.

For theta/beta and SCP training, the NF system Self-regulation and Attention Management (SAM; developed by our group) was used. Brain electrical activity (recorded via sintered Ag/AgCl electrodes) was calculated from Cz (reference: one mastoid, sampling rate: 250 Hz, bandwidth T/B: 1–30 Hz, bandwidth SCP: 0.01–30 Hz). Two additional EOG electrodes were placed above and below one eye in order to record blinks and vertical eye movements and the time course of the EOG channel was depicted on the trainer’s monitor. These ocular artifacts were corrected online using a regression-based algorithm (T/B: Semlitsch et al., 1986; SCP: Kotchoubey et al., 1997). When artifacts exceeded ±100 μV in the EEG channel or ±200 μV in the EOG channel, for these segments no feedback was provided to the subject.

#### Control training

The control training was no NF training and was only designed to parallel the transfer tasks included in the NF trainings (but not the amount of time) in order to control for both practice effects due to repeated testing (pre- and post-assessments) and for unspecific training effects related to developing and applying strategies to daily life situations. It comprised six sessions of about 20 min each which on average took place twice per week. Similar to the NF groups, before performing the transfer tasks (in turn a game of darts or a continuous performance test), subjects developed individual cognitive strategies that helped them to achieve an attentive state, a relaxed state or a state in which they were in a positive mood. Subjects were then instructed to activate these strategies before starting the transfer task. As in the NF groups, participants were instructed to practice their strategies in relevant daily life situations.

### LEARNING OF SELF-REGULATION SKILLS

Self-regulation of the theta/beta ratio during T/B training as well as differentiation between negativity and positivity trials during SCP training was analyzed. Self-regulation in the first two training sessions (average value of sessions one and two) was compared to self-regulation of the last two training sessions (average value of sessions nine and 10). Self-regulation measures presented here do not differentiate between trials with contingent feedback and transfer trials.

Associations of self-regulation abilities (good vs. poor performers) and pre–post changes in ERP measures (T/B: P3 amplitudes, SCP: CNV amplitudes) were calculated. For the analysis related to CNV amplitudes, self-regulation abilities were analyzed based on regulation abilities in negativity trials due to the close relation of negative SCPs and the CNV.

### ASSESSMENTS AND NEUROPHYSIOLOGICAL RECORDINGS

Participants of all three groups performed pre- and post-training assessments which took place before the start of training and in the week after the end of training, respectively. The laboratory assessments included the performance of an attention-demanding task while brain electrical activity was recorded, and a measurement with TMS.

#### Attention task and event-related potentials

As an attention-demanding task the Attention Network Test (ANT; Posner and Petersen, 1990; Fan et al., 2002) was selected. Subjects performed the ANT while brain electrical activity was recorded.

The ANT version used in the present study (Rueda et al., 2004) was realized in Presentation (Version 11.0; Neurobehavioral Systems, Albany, CA, USA) in a similar same way as described in Kratz et al. (2011) but with an additional variant including the presentation of a noise sound. The variant with the noise sound, in the following referred to as WithStress condition, was added in order to include a condition with higher demands. The test itself consisted of four blocks of 48 trials each, two blocks of each variant (with noise sound, without noise sound).

Subjects were presented five fish in a row (a middle fish surrounded by two flanking fish on each side) and were instructed to respond with a left- or right-mouse click depending on the direction in which the middle fish (target fish) was pointing. The target fish was presented 100 ms after the four flanking fish. Trials were congruent (resp. incongruent) if the fish flanking the middle fish were pointing in the same (resp. opposite) direction. Three cue conditions were included in the task and cues were presented 1400 ms before the target fish: no cue was presented (NoCue condition), a cue was presented in the center of the screen (NeutralCue condition), a cue was presented above or below the center of the screen, i.e., at the location where the target fish was to appear (SpatialCue condition). The performance measures number of hits, mean RT, and variability of RT (RTV).

EEG was recorded from 23 sites (10–20 system with FPz and Oz; recording reference: FCz; ground electrode: CPz; bandwidth: 0.016–120 Hz; sampling rate: 500 Hz) with sintered silver/silver-chloride (Ag/AgCl) electrodes and Abralyt 2000 electrolyte using the BrainAmp amplifier (Brain Products, Munich, Germany). In addition, vertical and horizontal EOG were recorded. Impedances were kept below 20 kΩ.

The data were analyzed with the Vision Analyzer software (Brain Products, Munich, Germany). Encephalogram was down-sampled to 256 Hz, re-referenced to the mastoids, and filtered oﬄine (resting EEG: 0.1–30 Hz, ERPs: 0.05–30 Hz; 12 dB/octave Butterworth filter; 50-Hz notch filter). Occular artifacts were corrected using the Gratton and Coles algorithm (Gratton et al., 1983). If EEG amplitude exceeded ±80 μV at any electrode a section of -500 to +500 ms around the artifact was removed in all channels. For the analysis of the interval between cue and target presentation, segments of 1800 ms length were formed, which started 230 ms before cue presentation. The CNV was determined at Cz as the mean amplitude in the time window 1000–1300 ms after cue onset. Target processing was analyzed based on segments of 1250 ms length, which started 125 ms before target presentation. The P3 was determined as the most positive peak at Pz in the time window 280–450 ms after target presentation. For ERP analysis, only trials with correct responses were considered and averaged responses of a participant were required to be based on at least 20 artifact-free segments. In order to avoid distortion of the ERP topography, no baseline correction was applied.

#### TMS

Transcranial magnetic stimulation measurements based on the double-pulse paradigm (Kujirai et al., 1993) were performed, while subjects remained in a resting state. Electromyogram (EMG) activity was recorded at the musculus abductor digiti minimi of the right hand.

For the TMS measurements, recording settings of the amplifier were adjusted accordingly (bandwidth: 8–1000 Hz, sampling frequency: 5 kHz). A figure-of-eight coil (diameter of one wing: 70 mm) connected to a Magstim Bistim unit with two Magstim 200^2^ stimulators (Magstim, Whitland, UK) was used for the measurements. The stimulation position was determined as the position of the coil on the scalp which elicited the largest motor evoked potential (MEP). The resting motor threshold (RMT) was determined as the minimal stimulus intensity that did not elicit an MEP larger than 50 μV in five consecutive trials. The suprathreshold stimulus intensity was determined such that MEP amplitude was about 1 mV (peak-to-peak) and the intensity of the conditioning stimulus was set to 75% of RMT. During measurement, paired pulses were used for stimulation which consisted of the conditioning stimulus followed by the suprathreshold stimulus. The inter-stimulus interval of these two pulses was set to 2, 3, 4, or 5 ms for inhibitory trials and to 7, 9, 12, or 18 ms for facilitatory trials. The task consisted of 50 trials that were pseudo-randomized in blocks of five trials, which consisted of a single-pulse trial (without a conditioning stimulus), two inhibitory and two facilitatory trials. The task was performed twice with a short break in between.

Data were segmented into trials. If in a time window of 40 ms before stimulation, peak-to-peak amplitude exceeded 45 μV, this trial was discarded due to initial muscle tension. The MEP amplitude was determined as the peak-to-peak amplitude of the most positive and most negative peak in a window of 65–100 ms after stimulation. If the MEP amplitude of the single-pulse trial was below 400 or above 2000 μV, the whole block of five trials related to this single-pulse trial was discarded. The relative MEP amplitude was determined by dividing the MEP amplitudes of double-pulse trials by the MEP amplitude of the single-pulse trial of the corresponding block of five trials. For inhibitory and facilitatory trials, the average relative MEP was calculated per subject reflecting SICI and ICF, respectively. A subject was excluded from further analysis, if less than 14 trials with sufficient data quality remained for inhibitory or for facilitatory trials in the pre or post measurement.

### STATISTICAL ANALYSIS

Statistical data analysis was performed using the software PASW Statistics (v.18). Repeated-measure ANOVAs with the between-subject factor GROUP (T/B, SCP, CON), the within-subject factor TIME (pre, post) were performed for all measures. For all ANT analyses, an additional within-subject factor STRESS (NoStress, WithStress) was included. For the CNV analysis, a factor CUE (NeutralCue, SpatialCue), and for the target-P3 analysis a factor CUE (NoCue, NeutralCue, SpatialCue) were added (as in the NoCue condition no CNV is elicited, this condition had to be excluded for the CNV analysis). Results were reported if at least a trend was revealed in the ANOVA.

Statistical analyses were based on data for which extreme values (larger/smaller than 2.5 standard deviations) had been excluded. For the self-regulation analyses, extreme values were not excluded due to very small group sizes resulting from the application of a median split.

An exploratory analysis was performed based on pre–post change scores between groups. Effect sizes (Cohen’s *d*) were reported where at least medium effect sizes were revealed. Effects were interpreted following the notion that *d* = 0.20 indicates a small, *d* = 0.50 a medium, and *d* = 0.80 a large effect (Cohen, 1988).

In addition, for all-measures ANOVAs were calculated for pre-training data and results were only reported if significant pre-training differences were observed.

Self-regulation analyses performed for the SCP and theta/beta groups were based on Student’s *t*-tests. As we had directed hypotheses regarding associations of SCP negativity regulation and CNV amplitudes, one-sided, *t*-tests were applied. For the associations of theta/beta self-regulation and P3 amplitudes two-sided, *t*-tests were used, as we did not have directed hypothesis regarding the direction of P3 amplitude changes.

## RESULTS

### LEARNING OF SELF-REGULATION SKILLS

**FIGURE 1:**
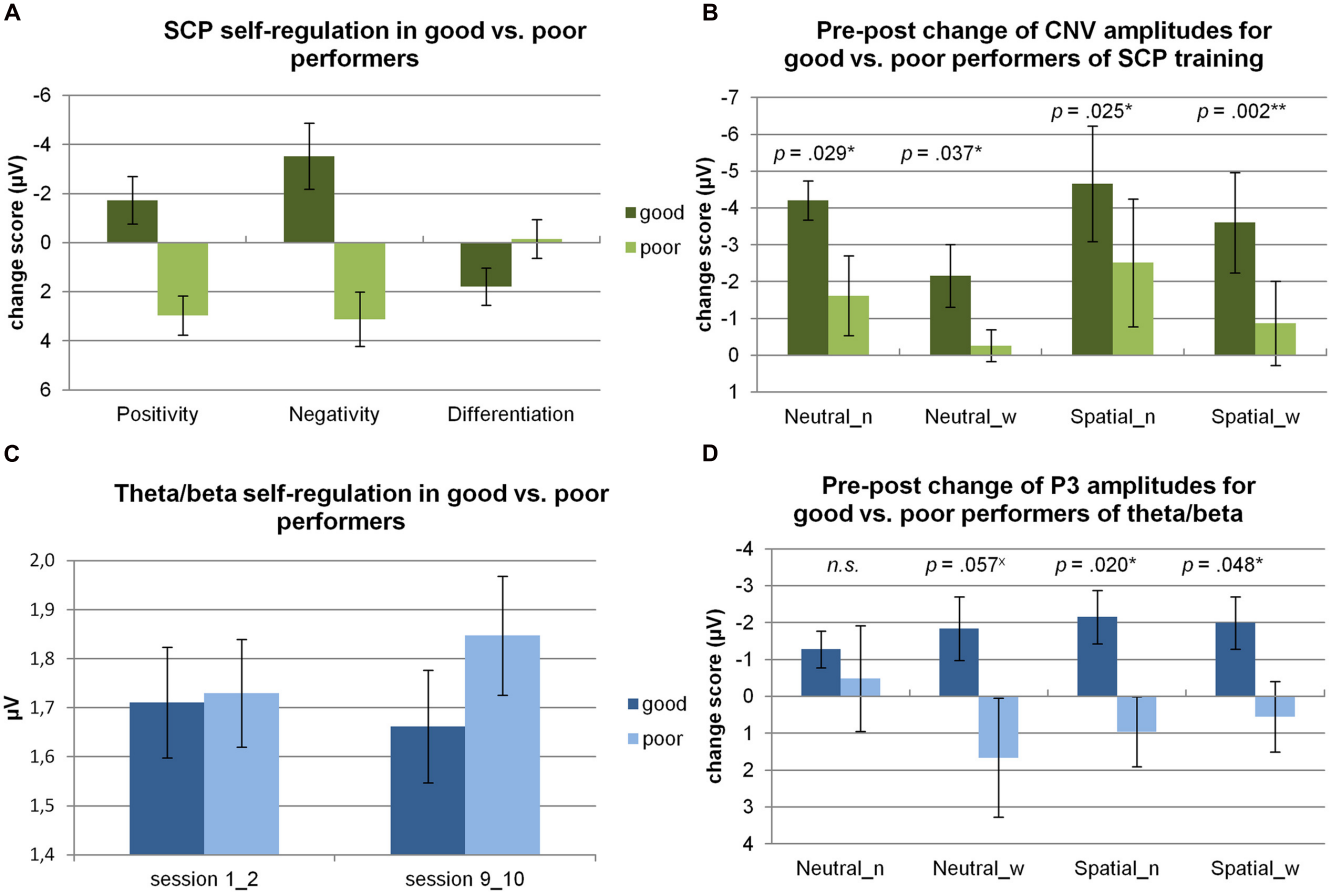
**Self-regulation skills. (A)** Changes in SCP self-regulation skills from the beginning (first two double sessions) to the end of training (last two double sessions) are depicted for good vs. poor performers. Self-regulation is shown for positivity and negativity trials, and for the differentiation between positivity and negativity trials. The group of good and poor performers is based on the median split of negativity regulation performance. **(B)** Pre**–**post changes in CNV amplitudes (at Cz) are depicted for good vs. poor performers of negativity regulation for the NoStress (n) and WithStress (w) conditions during NeutralCue and SpatialCue trials. In addition, *p* values of the one-sided, Student’s *t*-tests performed to compare CNV amplitudes of good and bad performers are depicted. **(C)** Changes in theta/beta self-regulation skills (theta/beta ratio) from the beginning (double sessions 1 and 2) to the end of training (double sessions 9 and 10) are depicted for good vs. poor performers based on the median-split of theta/beta ratio self-regulation. **(D)** Pre**–**post changes in target-P3 amplitudes (at Pz) are depicted for good vs. poor performers of theta/beta regulation for the NoStress (n) and WithStress (w) conditions during NeutralCue and SpatialCue trials. In addition, *p* values of the two-sided, Student’s *t*-tests performed to compare P3 amplitudes of good and bad performers are depicted. ^x^trend; *significant result, i.e., *p* < 0.05, **result remains significant after Holm–Bonferroni correction due to multiple comparisons.

For the SCP group (*n* = 17), a trend was obtained for a change in differentiation from the beginning to the end of training (pre: *M* = -1.02 μV, SD = 1.43 μV, post: *M* = -0.20 μV, SD = 2.63 μV; *t*(15) = -1.40, *p* < 0.10, Cohen’s *d* = 0.32). When comparing the change in self-regulation of good and poor performers from the beginning to the end of training (see **Figure 1A**), good performers based on negativity self-regulation during SCP training were able to produce, e.g., significantly more pronounced negative potential shifts in the course of training than poor performers (*t*(14) = 3.81, *p* < 0.01).

For theta/beta training (*n* = 16), theta/beta ratio did not significantly change in the course of training (pre: *M* = 1.72, SD = 0.31, post: *M* = 1.75, SD = 0.34; *t*(16) = -1.05, n.s.). When comparing the change in self-regulation of good and poor performers from the beginning to the end of training (see **Figure 1C**), a significant difference was obtained for good compared to bad performers based on self-regulation of the theta/beta ration during theta/beta training (*t*(15) = 4.14, *p* = 0.001).

### ATTENTIONAL PROCESSES

#### Performance measures

With respect to attention as measured by the ANT (see **Table 2**), RT (*n* = 51) significantly decreased from pre- to post-assessment [TIME: *F*(1,48) = 15.58, *p* < 0.001] and training type showed a tendency to have an effect on this pre–post decrease in RT [TIME × GROUP (*F*(2,48) = 2.84, *p* < 0.10)]. These group differences were mainly related to larger decreases in the T/B group in the range of medium to large effect sizes (see **Table 3**).

**Table 2:** Attention Network Test performance.

	Theta/beta		SCP		Control	
	Pre	Post	Pre	Post	Pre	Post
Hits_n	95.0 ± 1.2	95.4 ± 0.7	94.8 ± 1.1	94.9 ± 1.6	94.4 ± 1.4	95.1 ± 0.9
Hits_w	94.9 ± 0.9	94.8 ± 1.4	94.5 ± 1.2	94.6 ± 1.2	94.5 ± 1.5	94.6 ± 1.6
RT_n (ms)	426.1 ± 32.1	400.2 ± 33.3	415.1 ± 41.4	408.9 ± 46.4	419.1 ± 36.8	404.5 ± 29.2
RT_w (ms)	417.2 ± 32.8	392.4 ± 27.7	407.4 ± 39.2	402.8 ± 43.7	409.5 ± 36.1	401.5 ± 29.9
RTV_n (ms)	72.8 ± 16.9	60.0 ± 19.8	75.6 ± 22.1	62.7 ± 23.9	74.0 ± 19.7	67.1 ± 15.8
RTV_w (ms)	64.4 ± 14.0	55.9 ± 9.6	63.3 ± 15.2	64.4 ± 21.3	69.8 ± 20.7	64.6 ± 20.0

*For each group, the mean score (±SD) of each measure of the ANT are depicted at both pre- and post-assessment. SCP, slow cortical potential training group; RT, reaction time; RTV, variability of reaction time; ms, milliseconds; n, NoStress condition; w, WithStress condition.*

**Table 3:** Effect sizes (Cohen’s *d*).

	Theta/beta vs. control	SCP vs. control	Theta/beta vs. SCP
**Attention Network Test (ANT)**			
Reaction time (RT) total score	**0.61**	-0.21	**0.82**
**Contingent negative variation (CNV)**			
NoStress/WithStress	**0.66/1.01**	**0.57/0.84**	0.00/0.06
**Transcranial magnetic stimulation (TMS)**			
SICI	|**1.08**|	| 0.44|	**| 0.65|**
ICF	|-0.17|	**|-0.78|**	**| 0.64|**

*Effect size measures (Cohen’s *d*) are depicted for the comparison of pre–post change scores between groups. Positive values of effect sizes indicate a larger improvement (or smaller decline) in the group mentioned first compared to the group mentioned second. Black numbers indicate small effect sizes, black bold numbers medium effect sizes, and black bold underlined numbers large effect sizes, while gray numbers indicate no effect. SCP, slow cortical potential training group; total score: based on data averaged over NoStress and WithStress conditions; TMS: for this measure effect sizes are depicted in brackets since it is not clear a change in which direction constitutes an improvement; SICI, short-interval intracortical inhibition; ICF, intracortical facilitation.*

Regarding the number of correct responses (*n* = 48), no significant pre–post changes were observed [TIME: *F*(1,45) = 2.43, n.s.; TIME × GROUP: *F*(2,45) = 0.42, n.s.]. While the variability of RTs (*n* = 51) significantly decreased from pre to post [TIME: *F*(1,48) = 9.23, *p* < 0.01], no significant effect of training type could be observed [TIME × GROUP: *F*(2,48) = 0.41, n.s.].

For the performance measures, no group-specific effects including the factor STRESS were observed.

#### CNV

**FIGURE 2:**
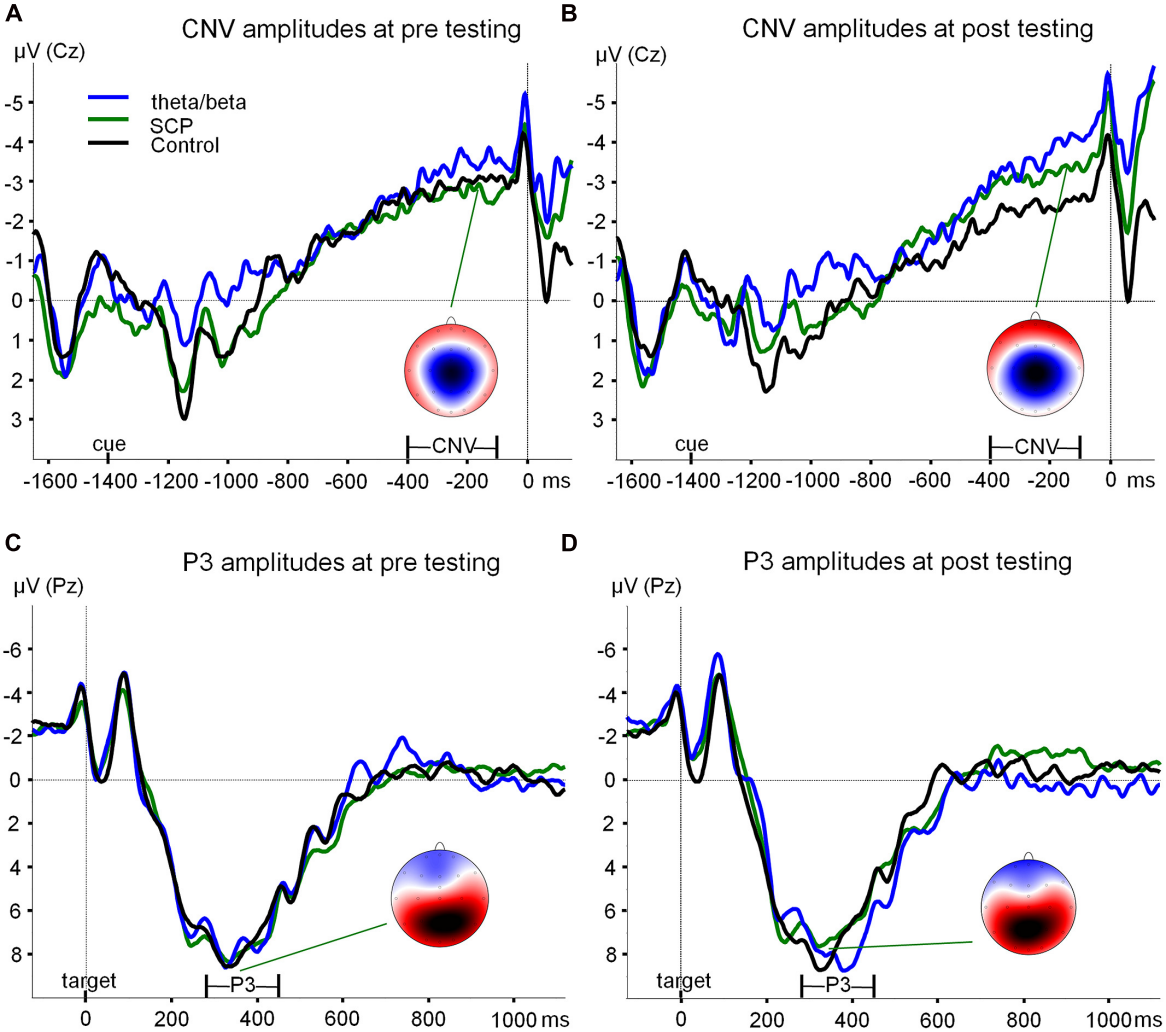
**ERPs during the ANT. (A)** Grand average ERPs (at Cz) during the preparation phase in the ANT at pre-testing are depicted exemplary for SpatialCue trials (averaged over the NoStress and WithStress conditions) for each training group (theta/beta: blue line, SCP: green line, control: black line). At -1400 ms a cue was presented, flanking fish appeared at -100 ms, and the target fish appeared at 0 ms. Contingent negative variation was determined as the mean area between -400 and -100 ms. Spline-interpolated maps illustrate the topography of the CNV exemplary for the SCP group, with blue and red colors indicating negative and positive amplitude values, respectively in a range from -4 to 4 μV. **(B)** Grand average ERPs (at Cz) during the preparation phase in the ANT at post-testing. **(C)** Grand average ERPs (at Pz) during target processing in the ANT at pre-testing are depicted exemplary for SpatialCue trials (averaged over the NoStress and WithStress conditions) for each training group (theta/beta: blue line, SCP: green line, Control: black line). At 0 ms, the target fish appeared. P3 amplitude was determined as the most positive peak at Pz in the time window 280–450 ms after target presentation. Spline-interpolated maps illustrate the topography of the P3 exemplary for the SCP group, with blue and red colors indicating negative and positive amplitude values, respectively in a range from -8 to 6 μV. **(D)** Grand average ERPs (at Pz) during target processing in the ANT at post-testing. SCP, slow cortical potential training group; CNV, contingent negative variation; ANT, Attention Network Test.

Grand average ERPs during the preparation phase of the ANT are depicted in **Figures 2A,B**. A significant interaction of TIME and GROUP was obtained [*F*(2,42) = 3.89, *p* < 0.05] indicating that type of training differentially affected attentional processing during anticipation as measured by CNV amplitudes during the ANT. This effect was related to a pre–post increase in CNV amplitude in both NF groups and a decrease in the control group. Effect size measures revealed medium to large effects for the T/B vs. CON and for the SCP vs. CON group, but no effect for the SCP vs. T/B group (see **Table 3**).

In addition, a significant effect of GROUP was observed [*F*(2,42) = 3.61, *p* < 0.05] which was related to higher overall CNV values in the SCP group.

No group-specific effects including the factor STRESS were observed.

In line with our hypotheses, good compared to poor negativity regulation during SCP training was associated with significantly larger pre–post increases of CNV amplitudes for all four stress/cue conditions according to one-sided, Student’s *t*-tests (see **Figure 1B**). Cohen’s *d* revealed large effects for all four conditions (NeutralCue_NoStress: *d* = 1.24; NeutralCue_WithStress: *d* = 1.15; SpatialCue_NoStress: *d* = 1.30; and SpatialCue_WithStress: *d* = 2.17).

#### Target-P3

Grand average ERPs during target processing in the ANT are depicted in **Figures 2C,D**. Attentional resource allocation during target processing as measured by target-P3 amplitudes did not significantly change from pre- to post-training [TIME: *F*(1,40) = 0.02, n.s.; TIME × GROUP: *F*(2,40) = 0.56, n.s.].

Regarding self-regulation of the theta/beta ratio, good performance was associated with significantly larger pre–post decreases of target-P3 amplitudes in the SpatialCue_WithStress and SpatialCue_NoStress condition and with a trend in the NeutralCue_WithStress condition, but not in the NeutralCue_NoStress condition according to two-sided, Student’s *t*-tests (see **Figure 1D**).

Cohen’s *d* revealed large effects for both SpatialCue conditions (NoStress: *d* = 1.38, WithStress: *d* = 1.14) and for the NeutralCue_WithStress condition (*d* = 1.05), but not for the NeutralCue_NoStress condition (*d* = 0.29), indicating large pre–post decreases in P3 amplitudes in good compared to poor performers in three out of four task conditions.

### MOTOR SYSTEM EXCITABILITY

**FIGURE 3:**
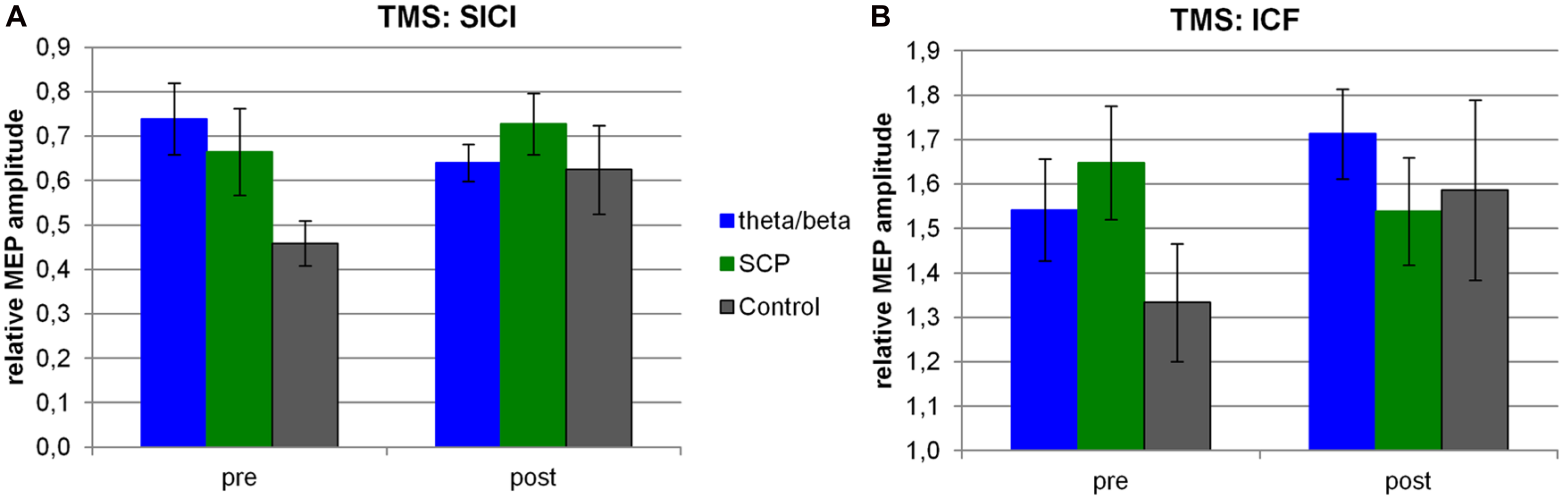
**TMS.** For each group, the relative MEP amplitude is depicted for SICI **(A)** and ICF **(B)** measures both at pre- and post-assessment. SCP, slow cortical potential training group.

For safety reasons, TMS measurement had not been performed in all subjects and data quality was not sufficient in some subjects which was related to the high variability of single-pulse MEP amplitudes. Thus, 28 subjects (T/B: *n* = 10, SCP: *n* = 9, CON: *n* = 9) could be included in further analyses (for more information see Studer, 2011). Relative MEP amplitudes for SICI and ICF measures are depicted in **Figure 3**.

The repeated-measure ANOVA calculated for the SICI measure resulted in a trend for the interaction of TIME × GROUP [*F*(2,25) = 2.83, *p* < 0.10]. This result was mainly related to differences between the T/B and the CON group, for which a large effect size was obtained and also to differences between T/B and SCP for which a medium effect size was observed (see **Table 3**).

A trend for pre-training group differences was obtained [GROUP: *F*(2,25) = 3.37, *p* < 0.10], related to higher SICI in the control group at pre-training.

Regarding ICF, no significant change from pre- to post-training was observed [TIME: *F*(1,25) = 1.49, n.s.; TIME × GROUP: *F*(2,25) = 1.52, n.s.]. Effect sizes for this ICF measure revealed medium effects for SCP vs. CON, and for T/B vs. SCP (see **Table 3**).

## DISCUSSION

The present randomized, controlled investigation in “healthy” adult participants aimed at examining the specificity of the effects of a theta/beta, and an SCP NF training on attention both at the performance and neurophysiological level (ERPs) as well as on motor system excitability (TMS). To our knowledge, the present study was the first study to examine motor system excitability by means of TMS after a complete NF training, and it was one of few studies which has examined the effects of SCP training in “healthy” adults and which has examined the neurophysiological mechanisms mediating the NF effects of both theta/beta and SCP training in a controlled design.

### LEARNING OF SELF-REGULATION SKILLS

Self-regulation skills of the theta/beta ratio as well as differentiation between negativity and positivity trials were not sufficiently learned in our study. This constitutes a limitation of our study and needs to be considered for the interpretation of the results. At the same time, regarding the self-regulation analysis, methodological aspects concerning training design and self-regulation analysis need to be considered and self-regulation results of our study need to be discussed in the light of NF literature.

One reason for the non-sufficient learning of self-regulation may be related to the training design of double sessions, which lasted for about two hours, which is much longer than the design commonly applied in adult NF studies. In addition, many of the participants had very packed time schedules and the demands of being attentive for such a long time may have been too long. This is supported by observations of the trainers that many participants became very tired in the course of training. In addition, the number of training sessions (10 double sessions) may not have been sufficient to acquire reliable self-regulation and the instruction of using cognitive strategies may have interfered with the processing of the contingent feedback signal.

Regarding methodological aspects of self-regulation analysis, so far there is no standardized analysis method in the literature. In our training design, within the first two sessions, the duration of most self-regulation blocks was much shorter than for the following sessions in order to allow subjects so accommodate to the training programs. Thus, self-regulation during shorter self-regulation blocks at the beginning of the training was compared to self-regulation during longer self-regulation blocks at the end of the training which required keeping up successful self-regulation for a longer period in a row.

In children with ADHD, some studies have reported on self-regulation abilities. Self-regulation abilities were acquired during theta/beta and SCP training (Leins et al., 2007), and learned self-regulation in the course of SCP training was associated with larger reductions in ADHD symptomatology in two studies (Strehl et al., 2006; Drechsler et al., 2007). However, comparability to our study remains limited as applying these NF protocols in patients may leave more room for improvements. Also the theta/beta training was realized in a different way including much shorter trials, a continuous updating of the baseline and activation as well as deactivation blocks.

Regarding studies in healthy adults, with respect to self-regulation abilities across sessions, mixed results are reported in the literature for different NF protocols (e.g., Raymond et al., 2005b; Doppelmayr and Weber, 2011; Weber et al., 2011; De Zambotti et al., 2012). In a comprehensive review of self-regulation abilities acquired in healthy adults, it is concluded that SMR learning has mainly been successful (Gruzelier, 2014), e.g., SMR learning was observed after 30 sessions by Doppelmayr and Weber (2011), but in a recent study by Gruzelier et al. (2014), across-session SMR learning was only observed by linear trends, and it was not observed in a study by Vernon et al. (2003). So far, SCP training has hardly been applied in healthy adults, but it has been shown, that adults are able to learn self-regulating their SCPs (Birbaumer, 1999). Regarding theta/beta training, Doppelmayr and Weber (2011) did not observe theta/beta theta/beta (4.5–7.5/17–21 Hz) learning after 30 training sessions, while they observed SMR learning in the SMR training group. Due to the differences in training protocols with respect to, e.g., frequency bands (we used a broader beta band ranging from 13 to 20 Hz), training implementation (duration of training sessions, including transfer trials, using cognitive strategies for self-regulation) limits comparability of the described results in addition to differences in the parameterization of self-regulation.

Despite the discussed limitations related to self-regulation, the study was able to indicate both some effects at the group level and differential effects between good and poor performers and thus indicating some specific effects of theta/beta and SCP training.

### ATTENTIONAL PROCESSES

Regarding attentional performance during the ANT, our study did not reveal any advantages of NF on the number of correct responses, mainly due to ceiling effects as performance in all groups was very good at pre-testing (e.g., nearly 100% correct responses). Faster responding and lower variability of responses during the ANT were observed over all groups, which constitute general learning effects related to the repeated task performance.

More specifically, a trend for a larger increase in response speed which was related to the type of training was observed. Effect sizes measures revealed a large effect for the theta/beta compared to the SCP group indicating a specific effect for theta/beta training.

While such an effect was not observed to be specific for theta/beta training in children with ADHD (Wangler et al., 2011), comparability of this finding to studies in healthy adults remains difficult as we used a broader beta band (13–20 Hz) and also due to the non-consistent findings in the literature. While faster responding in attention tasks was observed after beta training (15–18 Hz) and reduced variability of responding after SMR training (Egner and Gruzelier, 2004), a more recent study reported faster responding only after SMR but not after theta/beta (4.5–7.5/17–21 Hz) training (Doppelmayr and Weber, 2011). These mixed results may be related to differences in training protocols as has been discussed above, as well as to the different attention tasks employed in the different studies.

On the neurophysiological level, attentional resource allocation during the preparation phase of the ANT was improved after NF compared to CON training as indicated by medium to large effect sizes for each of the NF groups compared to the CON group. Contrary to our hypotheses, no differential effect between the SCP and theta/beta training groups was obtained. However, self-regulation abilities of good performers during SCP training were associated with a larger increase in CNV amplitudes compared to poor performers. In addition, it has to be taken into account that overall CNV amplitudes were highest in the SCP group (sign. effect for GROUP), and that despite higher CNV amplitudes at pre-assessment a pre–post increase in CNV amplitudes comparable to the one after theta/beta training was observed for SCP training. Overall, these results indicate a small specific effect for SCP training on attentional resource allocation as measured by CNV amplitudes.

Our findings of some specific effects of SCP training are in line (but less pronounced) with those of Wangler et al. (2011) who, in children with ADHD, have found the pre–post increase in CNV amplitude to be specific for SCP training. In children with ADHD, increased CNV amplitudes after SCP training compared to a waiting-list group had also been reported previously (Heinrich et al., 2004). Even though in a study by Doehnert et al. (2008) in children with ADHD a decrease in CNV amplitudes was observed after both SCP training and group therapy, this decrease was less pronounced in those children who successfully learned SCP self-regulation. Also in adults with ADHD, preliminary results after 15 SCP sessions indicated a trend toward a CNV amplitude increase (Mayer et al., 2012a,b). However, it has to be considered that in several studies in children with ADHD (Sartory et al., 2002; Banaschewski et al., 2003), and also in adults with ADHD (Mayer et al., 2012a,b), reduced CNV amplitudes have been observed compared to normal controls, which may have left more room for improvement than in “healthy” adults. Overall, in line with previous literature our results provide further evidence for specific effects of SCP training on resource allocation as assessed by CNV amplitudes.

Our findings of no overall pre–post change in P3 amplitudes after NF fits into the mixed results reported in the literature. No change in P3 amplitudes has been observed after an SMR training, while an increase in P3 amplitudes was observed after a beta1 (15–18 Hz) training in healthy adults (Egner and Gruzelier, 2004), and after an SMR training in six patients with ADHD who were considered responders of SMR training (Arns et al., 2012). As already discussed in a previous section, comparability of the results of the different studies is limited by the differences in NF protocols that were used as well as by the different attention tasks during which P3 amplitudes were assessed.

Based on self-regulation analysis measures, in our study a specific effect of theta/beta training on attentional resource allocation as assessed by P3 amplitudes was observed. Good performance during theta/beta training (theta/beta ratio) was to some extent (for some but not for all cue conditions) associated with reduced target-P3 amplitudes. Our results were in contrast to Egner and Gruzelier (2001) who observed regulation abilities of SMR as well as beta training in healthy adults to be positively correlated with increased P3 amplitudes. However, it remains to be questioned in how far larger P3 amplitudes are indicators of improved processing abilities. In children with ADHD, target P3 amplitudes during the ANT were observed to decrease from pre- to post-training (combined theta/beta and SCP NF or attention skills training) while at the same time performance improved and in addition, larger decreases in P3 amplitudes after training were reported for more intelligent children (Wangler et al., 2011). Moreover, repeated task performance had been associated with decreased P3 amplitudes (Howells et al., 2010). Thus, the hints for decreased P3 amplitudes observed after theta/beta training in our study may also be seen as indicating more efficient stimulus processing.

In summary, in our study differential effects of theta/beta and SCP training on attention were less pronounced than expected. While increased attentional resource allocation was observed for both NF protocols compared to the control group, successful SCP regulation was associated with increased CNV amplitudes suggesting a specific effect for SCP training. Theta/beta training was associated with a larger increase in response speed and successful theta/beta regulation was associated with reduced P3 amplitudes suggesting a specific effect of theta/beta training on more efficient stimulus processing. These results can be seen as in line with the neurobehavioral model of NF (Gevensleben et al., 2012).

### MOTOR SYSTEM EXCITABILITY

Regarding motor system excitability, our TMS results after a complete NF training schedule did not constitute an extrapolation of the TMS effects after a single-session NF study by Ros et al. (2010), which had also been performed with different NF protocols, but rather indicated a different pattern of results.

Our study revealed a trend for training effects on SICI, which was related to an increase in SICI after theta/beta training as indicated by a large effect size for the T/B vs. CON and by a medium effect size for T/B vs. SCP group. Thus, our data suggest a specific effect of theta/beta training on increasing SICI. This constitutes an interesting finding as the motivation for studying effects of NF on motor system excitability was derived from the application of NF training in children with ADHD. Reduced SICI is a common finding in ADHD literature, and methylphenidate has been reported to increase SICI in children with ADHD (Moll et al., 2002). Thus, in healthy adults, theta/beta training exerted similar effects on motor system excitability as methylphenidate in children with ADHD.

In an exploratory analysis solely based on effect size measures, our results suggested a specific effect of theta/beta training on increasing both SICI and ICF. A treatment leading to an increase in SICI in combination with an increase in ICF is a rare finding in the TMS literature. Kirschner et al. (2003) observed an increase of both SICI and ICF in healthy adults after a single-dose treatment with methylphenidate. Thus, in healthy adults, theta/beta training exerted similar effects on motor system excitability as methylphenidate.

However, limitations of the TMS analysis were the small group sizes, the trend for pre-training group differences for the SICI measure (trend for higher SICI in the CON group at pre-training) and results being mainly based on an exploratory effect size analysis. In addition, the functional significance of changes in motor system excitability during a resting state in healthy adults is not clear. Due to the small group sizes, the good–bad performer analysis based on theta/beta and SCP self-regulation could not be performed for the TMS measures.

Overall, our study was the first study to report effects of a complete NF training on motor system excitability. Changes in motor system excitability after theta/beta training paralleled the effects of methylphenidate in children with ADHD, i.e., an increase of SICI was observed. In an exploratory analysis, the increase in SICI and ICF observed after a theta/beta training also paralleled the effects of methylphenidate in healthy adults. Further research based on a larger sample is needed to validate these findings and studying motor system excitability during NF self-regulation may allow to better evaluate the functional significance of observed changes.

### METHODICAL ISSUES

The present investigation was conducted in “healthy” adults and not in children with ADHD due to the very comprehensive pre and post assessments, and in order to recruit a larger and more homogeneous sample. However, regarding the aim of a relatively homogeneous sample, it proofed difficult to recruit healthy adults who wanted to spend that much time for the comprehensive training sessions. Thus, adults with some kind of subclinical symptomatology (which according to the Symptom-Checklist-90 was more pronounced in the control group) were included in the study which may have affected the results.

The theta/beta protocol in our study included a broader beta band (13–20 Hz), which made comparability to some findings in healthy adults difficult, as in those studies training was based on separate and smaller SMR and beta bands. However, the theta/beta protocols used in our study has been successfully applied in children with ADHD and therefore can be considered a legitimate approach.

Regarding statistical analysis, due to the limited sample size medium effects did not reach the level of significance. A larger sample would have been needed in order to delineate robust results instead of reporting results based on effect size measures, despite the sample size of the present study being comparable to previous peak performance NF studies (e.g., Egner and Gruzelier, 2004; Ros et al., 2009; Logemann et al., 2010; Doppelmayr and Weber, 2011).

## CONCLUSION

Self-regulation skills were not sufficiently learned during theta/beta and SCP training, which needs to be considered as a limitation of our study. Yet, based on the good–poor performer analysis, some specific training effects on ERP components were observed. In line with the literature of NF in ADHD, our study provided further support for the SCP-specific effects on attentional resource allocation (CNV amplitudes) during response preparation also in “healthy adults.” Theta/beta training was associated with increased response speed and reduced attentional resource allocation (P3 amplitudes) during target processing, adding to the mixed results reported in both ADHD and peak performance literature. Moreover, motor system excitability measures suggested parallels of the effects of a theta/beta training to those of methylphenidate, constituting a new finding.

Future studies including larger sample sizes are needed to further evaluate the protocol-specific effects on attention and motor system excitability reported. Moreover, examining which factors mediate a more reliable acquisition of self-regulation skills, methodical issues of the parameterization of self-regulation as well as assessing motor system excitability during self-regulation can be considered as relevant topics for future research.

## Conflict of Interest Statement

The authors declare that the research was conducted in the absence of any commercial or financial relationships that could be construed as a potential conflict of interest.

## Footnotes

1 It has to be considered that the exact realization of the theta/beta protocol differs between research groups, e.g., with respect to the frequency range.
